# An Innovative Use of Maxillofacial Implants for the Open Reduction and Internal Fixation of a Displaced Sternal Fracture: A Case Report

**DOI:** 10.7759/cureus.76582

**Published:** 2024-12-29

**Authors:** Shinya Yamamoto, Hisanori Kani, Masakatsu Yamashita, Makoto Adachi

**Affiliations:** 1 General Surgery, Nagoya Tokushukai General Hospital, Kasugai, JPN; 2 Thoracic Surgery, Nagoya Tokushukai General Hospital, Kasugai, JPN; 3 Oral and Maxillofacial Surgery, Nagoya Tokushukai General Hospital, Kasugai, JPN

**Keywords:** chest trauma, intermaxillary fixation screw, maxillofacial implants, maxillofacial locking plate, open reduction and internal fixation, sternum fracture

## Abstract

Sternal fractures resulting from blunt chest trauma often present unique surgical challenges. While conservative management is common, cases with significant displacement, delayed union, or painful dyspnea may require surgical intervention to improve structural stability and relieve symptoms. Here, we report the case of a 46-year-old man who sustained a displaced sternal fracture in a motor vehicle accident. The fracture was complicated by posterior displacement of the proximal bone fragment, causing persistent chest pain and deformity that did not resolve with conservative treatment. The patient underwent open reduction and internal fixation (ORIF) using maxillofacial implants, including intermaxillary fixation (IMF) screws and maxillofacial locking plates. This innovative approach facilitated controlled reduction and stable fixation of the displaced fracture. A multidisciplinary team performed the surgery, highlighting the importance of collaboration in the management of complex chest injuries. Postoperative imaging confirmed anatomical alignment, and the patient reported significant relief of symptoms at one-month follow-up.

This case highlights the versatility and effectiveness of maxillofacial implants in the treatment of particularly challenging sternal fractures, such as chronic cases where traditional fixation methods are inadequate. The purpose of this report is to highlight the potential of maxillofacial implants as a safe and effective alternative to traditional methods in the treatment of complex sternal fractures and to encourage further research into their broader clinical application.

## Introduction

Sternal fractures are relatively rare injuries, with a reported incidence varying between different regions and healthcare systems. While Western studies indicate they account for 3%-8% of blunt chest trauma cases and 0.5% of all fractures [1,2], the most common mechanism of sternal fracture is injury to the anterior chest wall secondary to a motor vehicle crash. Over the past few decades, the incidence of sternal fractures has decreased with the widespread use of safety belts [3,4]. Furthermore, the majority of fractures occur in the body of the sternum, and displaced fractures are more frequently associated with chest injuries [5]. While most cases (>95%) are managed nonoperatively, complex fractures with large displacements, respiratory compromise, and delayed union often require surgical intervention [3]. This report describes a novel application of maxillofacial implants in the surgical management of displaced sternal fractures. Intermaxillary fixation (IMF) screws were useful in reducing displaced sternum fragments, and maxillofacial locking plates, originally designed for facial bone reconstruction, highlight their versatility and efficacy. Adapting these implants for sternal fixation may provide an alternative to traditional methods and widen the range of surgical options for chest trauma.

## Case presentation

**Figure 1:**
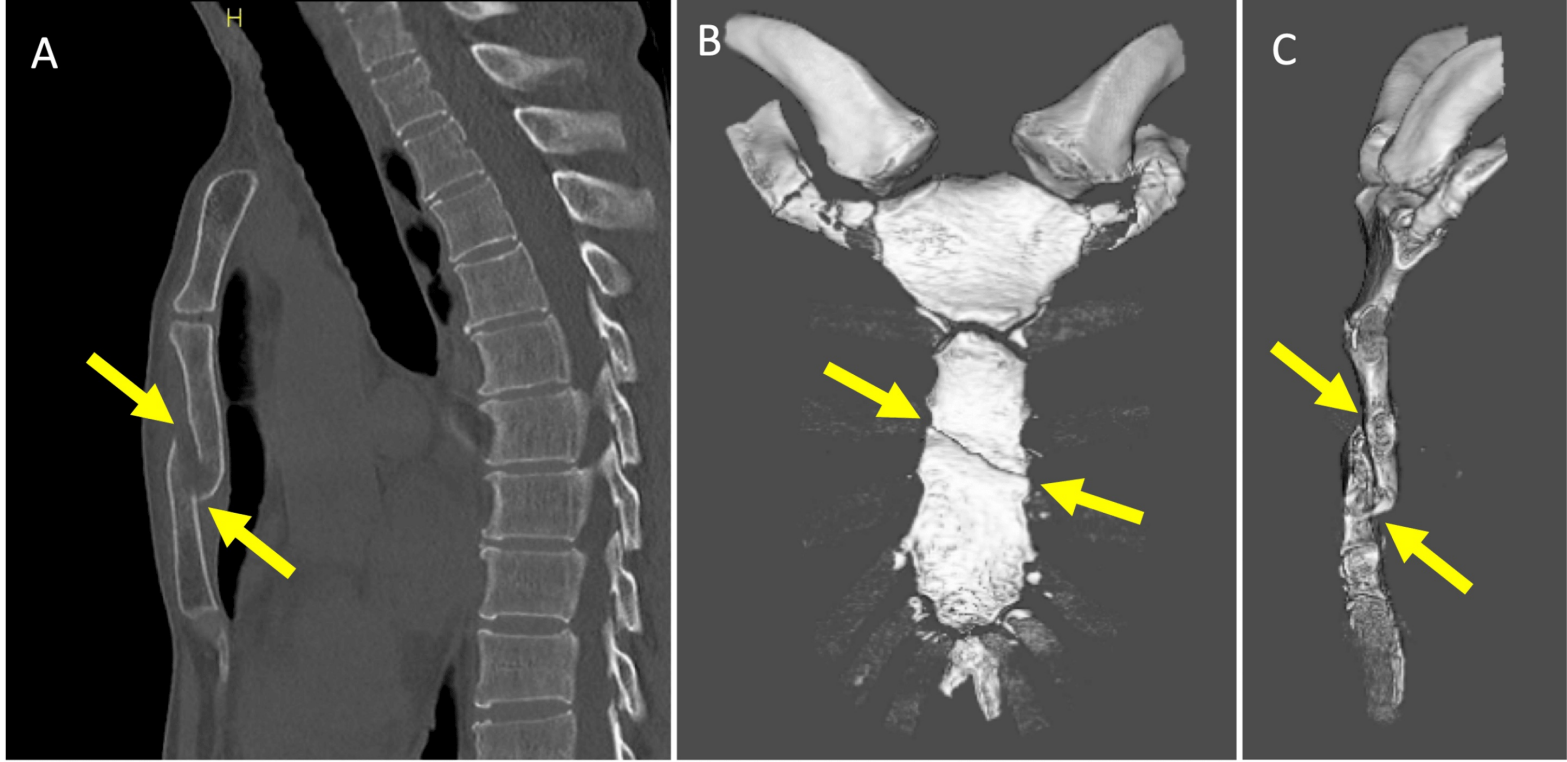
Preoperative imaging of sternal fracture (A) Sagittal CT image showing fracture site (arrow); (B) Anterior view of three-dimensional reconstructed CT image with fracture line (arrow); (C) Lateral view of three-dimensional reconstructed CT image with fracture location (arrow) CT, Computed tomography

A 46-year-old man was injured when he collided with a utility pole while driving a passenger car to avoid a motorized bicycle that had fallen forward. He was rushed to another hospital and initially diagnosed with a chest contusion, but one month later, he was referred to the Thoracic Surgery Department of our hospital due to persistent pain and deformation in the anterior chest. At the initial visit, sternal deformation was observed in the anterior chest, and he experienced sternal pain when coughing. One month later, he attended our hospital, complaining of persistent pain and chest deformity. Computed tomography (CT) imaging revealed a transverse sternal fracture of the sternum with displacement of the proximal fragment posteriorly (Figures 1A-1C).

**Figure 2:**
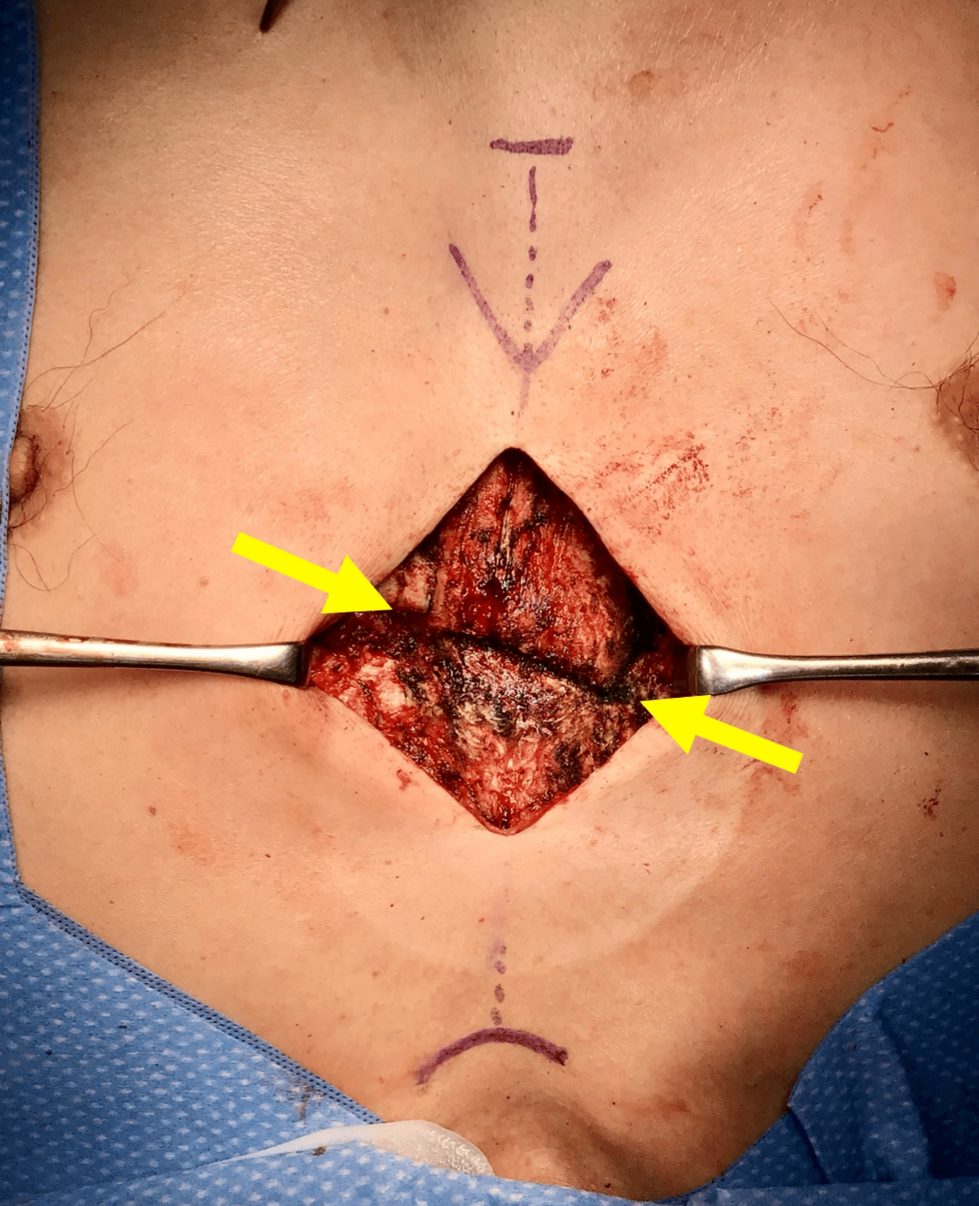
Intraoperative findings of exposed sternal fracture Posterior displacement of the proximal fragment is observed at the fracture line (arrow).

Due to chronic damage and ongoing symptoms, conservative treatment was deemed insufficient. The patient underwent open reduction and internal fixation (ORIF) of the sternum under general anesthesia two months after the injury. A team consisting of a thoracic surgeon, a general surgeon, and an oral and maxillofacial surgeon performed the surgery. A 10-cm midline incision was made in the anterior chest, and the sternum was exposed (Figure 2).

**Figure 3:**
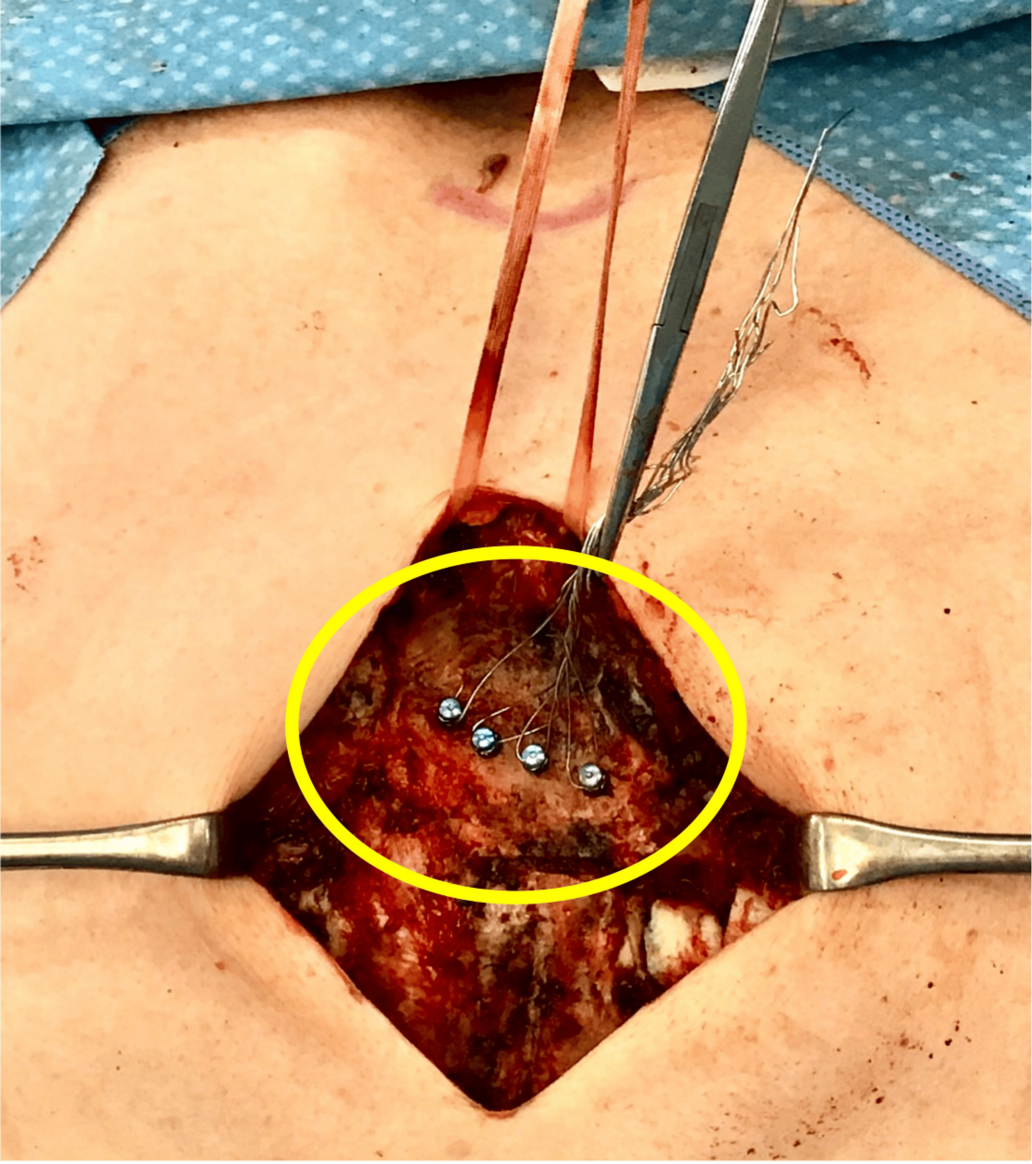
Application of IMF screws for fracture reduction Four IMF screws (circles) were placed and connected with wires for traction. IMF, Intermaxillary fixation

The fracture was difficult to reduce due to the chronicity of the injury. The proximal fragment was deeply displaced posteriorly, and elevation of the sternum was challenging. Reduction and fixation required careful manipulation to avoid damaging adjacent structures, such as the pericardium and ascending aorta. Reduction was achieved by inserting four IMF screws (TraumaOne IMF System; Zimmer Biomet, Warsaw, IN, USA) into the displaced bone fragments (Figure 3).

**Figure 4:**
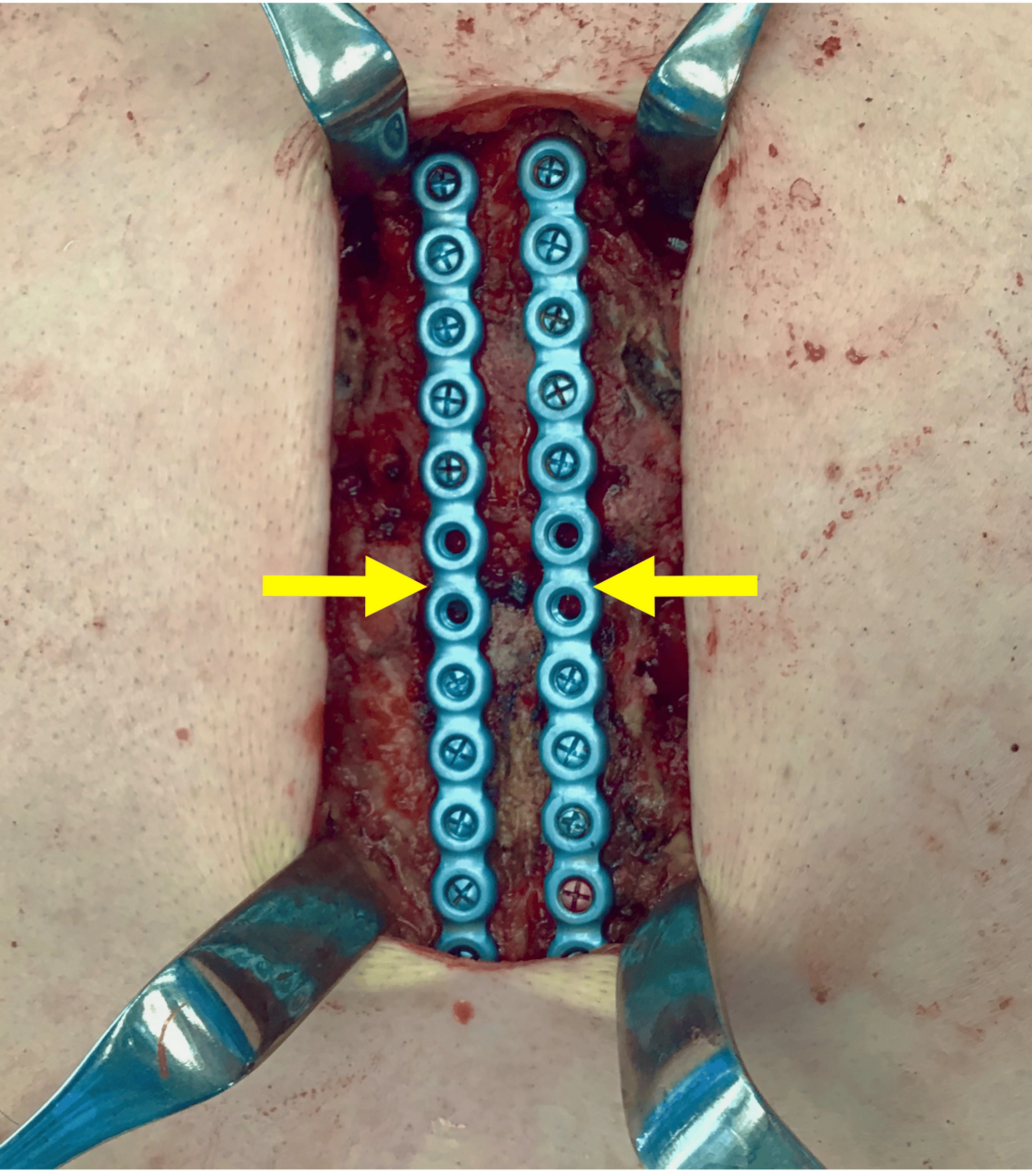
Fixation of the fracture using maxillofacial fixation plates Placement of two maxillofacial fixation plates (arrows).

The IMF screws were selected based on the anterior-posterior width measured in advance using CT images, and a screw length that would not penetrate the medial cortex of the sternum was selected. These screws secured the wires and facilitated traction and alignment control. Two titanium maxillofacial fixation plates (Matrix Mandible Plate System; Synthes, Warsaw, IN, USA) were then secured with screws to achieve fixation (Figure 4).

**Figure 5:**
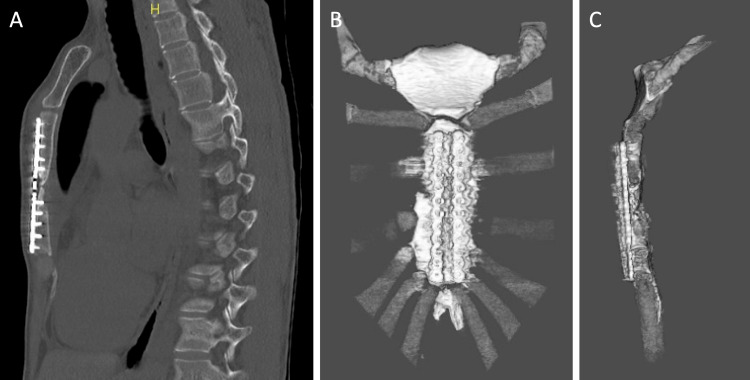
Postoperative images of sternal fracture (A) Sagittal CT image; (B) Anterior view of three-dimensional reconstructed CT image; (C) Lateral view of three-dimensional reconstructed CT image CT, Computed tomography

When drilling the maxillofacial locking plate, the IMF screws were used to manually create screw holes to prevent penetration of the medial cortex of the sternum. Postoperative CT imaging confirmed anatomical alignment and stable fixation, and the patient reported significant relief of symptoms at the one-month follow-up (Figure 5).

## Discussion

There is no consensus on the indications for surgical treatment of sternal fractures [6,7]. Patients undergoing surgical stabilization of sternal fractures had a lower mortality rate than those managed nonoperatively, highlighting the potential life-saving benefits of surgical intervention in appropriately selected cases [8]. Surgical fixation significantly reduced opioid requirements and improved respiratory function compared with nonoperative management, suggesting that surgery may enhance patient outcomes in selected cases [4]. The consensus warrants surgery in cases of significant displacement, persistent pain, respiratory distress, or delayed union that conservative management cannot resolve. In this case, the fractures were overlapping and significantly displaced, and we determined that the bone fragments would not heal with conservative treatment. Chronic displacement of sternal fractures often results in difficult fracture reduction due to remodeling and soft-tissue adhesions, necessitating a more innovative approach to achieve stable fixation [9].

Surgical fixation of sternal fractures requires careful attention to the underlying structures, including the pericardium, ascending aorta, and mediastinal tissues [10]. In this case, the proximal fragment was displaced posteriorly, requiring careful dissection around the fragment to prevent injury to critical structures. This underscores the importance of meticulous surgical planning and execution in managing sternal fractures.

Bone forceps were used to attempt to reduce the fracture, but we were unable to obtain sufficient purchase on the depressed fragment. The chronicity of the injury further complicated reduction, as the posterior displacement created significant mechanical challenges. The use of IMF screws allowed us to apply the necessary traction to the fragment, facilitating precise alignment and reduction of the fracture to the desired position.

Traditionally, sternal fractures have been fixed with stainless-steel wire. The advantages of stainless-steel wire are its low cost and ease of use [11]. However, there is a risk of secondary fracture when passing the wire through the sternum, particularly in osteoporotic or fragile bone [12]. Additionally, non-rigid fixation with stainless-steel wire can increase the risk of delayed union, which may lead to prolonged recovery and increased morbidity.

Plates and screws reduce the risk of these complications by providing rigid fixation, which promotes reliable healing [13]. One such system, SternaLock® (Biomet Microfixation Inc., Jacksonville, FL, USA), is specifically designed for sternal fractures. The advantages of SternaLock® include its thin profile and the ease of cutting the connecting portions on both sides. This facilitates a median sternotomy if needed and ensures safe use without damaging the organs behind the sternum, as it utilizes mono-cortical screws [14,15]. However, its drawbacks include the limited variety of plate designs and its configuration, which is not optimized for fixing transverse sternal fractures due to the length and screw hole arrangement. As a result, SternaLock® was deemed unsuitable for this case.

The maxillofacial locking plate system provides significant advantages over traditional fixation methods for sternal fractures [16]. Its durability and versatility are evident in the variety of plate lengths and screw hole configurations designed to accommodate different fracture patterns. An additional benefit is that, when a median sternotomy becomes necessary following sternal fixation with this system, the parallel alignment of the two plates ensures unimpeded access to the thoracic cavity through the median sternotomy.

In this case, our technique showed promising results, but this is a single-case experience, and larger studies are needed to verify the safety and efficacy of this technique. The long-term outcomes of maxillofacial implants in sternal fixation remain unknown, especially regarding implant stability and potential complications. Future prospective studies should compare this technique with traditional fixation methods and focus on both immediate and long-term outcomes. Recent studies have shown that surgical fixation is associated with a better quality of life compared with conservative treatment [17], suggesting the importance of developing innovative reduction and fixation techniques for complex sternal fractures.

## Conclusions

This case demonstrates the successful application of maxillofacial implants in the management of a challenging displaced sternal fracture. The combination of an IMF screw for reduction control and a maxillofacial locking plate for rigid fixation provided excellent stability and facilitated adequate healing. The technique has several advantages, including accurate reduction of the fracture, stable fixation, and adaptability to different fracture patterns. The good clinical outcome in our cases suggests that this approach could be particularly useful in complex sternal fractures where conventional fixation methods may be inadequate. Although further clinical studies are needed to validate these findings, our experience indicates that maxillofacial implants are a promising option in the surgical armoury for sternal fracture management.
